# Pneumorrhachis: an uncommon finding in patients with COVID-19

**DOI:** 10.1590/0037-8682-0095-2021

**Published:** 2021-04-12

**Authors:** Bruno Hochhegger, Juliane Nascimento de Mattos, Edson Marchiori

**Affiliations:** 1 Universidade Federal de Ciências da Saúde de Porto Alegre, Porto Alegre, RS, Brasil.; 2 Universidade Federal do Rio de Janeiro, Departamento de Radiologia, Rio de Janeiro, RJ, Brasil.

**FIGURE 1:**
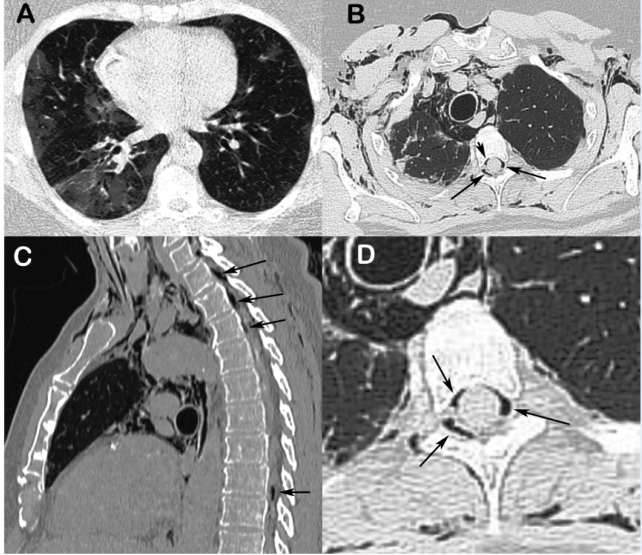
**(A)**, chest CT (axial reconstruction) showing right-sided predominant ground-glass opacities in both lungs. Chest CT with axial **(B)** and sagittal **(C)** reconstructions performed two weeks later, demonstrating subcutaneous emphysema, pneumomediastinum, and pneumorrhachis (arrows); **(D)**, spot film demonstrating a large amount of air within the spinal canal (arrows).

A 78-year-old man presented to the emergency department with a seven-day history of headache, fever, diffuse myalgias, dry cough, and dyspnea. Severe acute respiratory syndrome coronavirus 2 (SARS-CoV-2) infection was diagnosed by SARS-CoV-2 RNA detection in nasopharyngeal samples. Chest computed tomography (CT) demonstrated predominantly peripheral ground-glass opacities in both lungs (Figure 1A), suggestive of a viral infection. The patient’s cough markedly worsened during hospitalization. He experienced sudden onset anterior chest pain radiating to the neck, followed by dyspnea, after a severe coughing episode. The patient’s peripheral oxygen saturation on room air was 88%. Repeat CT showed extensive subcutaneous emphysema dissecting the muscular planes of the cervical and dorsal regions, extending into the mediastinum and medullary canal (Figures 1B-D). He was treated with analgesics, cough suppressants, and supplemental oxygen through a nasal cannula, showing partial improvement.

Pneumorrhachis (PR) is an uncommon condition defined as the presence of air in the spinal canal, most often resulting from spinal cord injuries or instrumentation; but also occasionally reported in association with spontaneous pneumomediastinum, as in our case. Spontaneous pneumomediastinum and PR can occur when intra-alveolar pressure increases, as occurs with forceful coughing, leads to the rupture of the central pulmonary alveolus. Air can move into the perivascular interstitium and dissect through the fascial planes from the posterior mediastinum or retropharyngeal space through the neural foramina into the epidural space^1^ ^-^ ^3^. Spontaneous pneumomediastinum-associated PR is usually self-limiting, following a generally benign, conservatively managed course.
